# HIV-1 Dynamics: A Reappraisal of Host and Viral Factors, as well as Methodological Issues

**DOI:** 10.3390/v4102080

**Published:** 2012-10-10

**Authors:** Heather A. Prentice, Jianming Tang

**Affiliations:** 1 Department of Epidemiology, University of Alabama at Birmingham, Birmingham, Alabama; Email: haprenti@uab.edu; 2 Department of Medicine, University of Alabama at Birmingham, Birmingham, Alabama; Email: jtang@uab.edu

**Keywords:** Correlates, genetics, HIV-1, viral load.

## Abstract

The dynamics of HIV-1 viremia is a complex and evolving landscape with clinical and epidemiological (public health) implications. Most studies have relied on the use of set-point viral load (VL) as a readily available proxy of viral dynamics to assess host and viral correlates. This review highlights recent findings from population-based studies of set-point VL, focusing primarily on robust data related to host genetics. A comprehensive understanding of viral dynamics will clearly need to consider both host and viral characteristics, with close attention to (i) the timing of VL measurements, (ii) the biology of viral evolution, (iii) compartments of active viral replication, (iv) the transmission source partner as the immediate past microenvironment, and (v) proper application of statistical models.

## 1. Introduction

HIV-1 infection typically occurs through a single viral variant [1,2,3,4,5], but the initial viral homogeneity is rather transient as the surviving viruses must mutate to evade host immune defenses or to regain fitness lost during adaptation to the immediate past host (the transmission source partner) [6]. At the population level, HIV-1 subtypes responsible for the global AIDS pandemic can vary by geographic region [7,8], while frequent superinfection can generate mosaic viruses (circulating recombinant forms) to promote viral diversity [8]. Understanding the evolution of HIV-1-host interactions requires close attention to both viral and host (immunologic) dynamics [9].

HIV-1 viral load (VL) set-point is a well-studied phenotype tied to virus-host equilibrium, with high set-point VLs translating to rapid disease progression [10,11,12,13,14,15,16,17] and fast transmission to susceptible hosts [18,19]. In many individuals, the viral ‘set-point’ is reached within weeks of infection [12,20,21], and it can remain relatively steady (±0.5 log_10_ RNA copies/ml) for years during clinical latency [10]. Progression to AIDS is usually accompanied by (i) rising VL, (ii) substantial loss of CD4+ T-cells in peripheral blood, and (iii) risk for opportunistic infections and malignancies. AIDS diagnosis based on < 200 CD4 cells/mm^3^ of blood and at least one opportunistic infection [22,23] can serve as another important phenotype for measuring the dynamics of host-virus interactions, but it can take close to a decade to develop even during untreated HIV-1 infection. In the era of highly active antiretroviral therapy (HAART), AIDS diagnosis is increasingly rare, so a focus on studying set-point VL as a proxy of viral fitness under specific microenvironment in the host is well justified, especially since many clinical decisions must be made during the early stages of HIV-1 infection [9,24].

**Figure 1:**
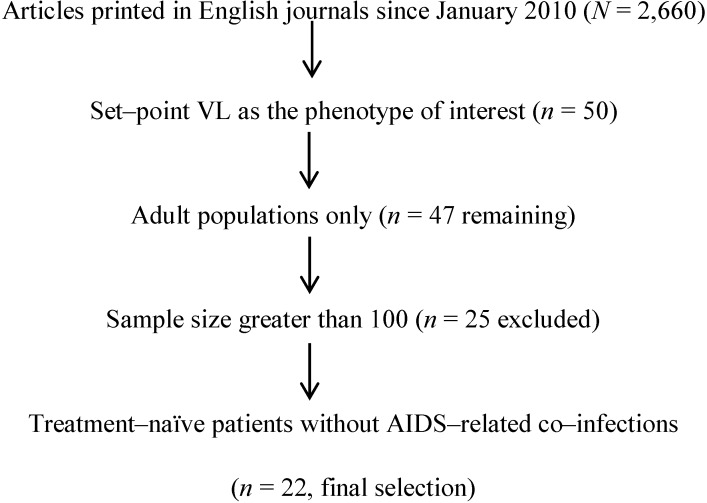
**Selection of recent (post-2010) publications for systematic review**. Two rounds of searches in PubMed yield 2,660 original research articles that contain three key words (HIV, viral load, and host or viral genome). Only 22 of these meet the criteria for full evaluation here (20 in Table 1 and two in Table 2).

HIV-1 VL was, in one way or another, a subject in over 2,600 articles published since January 2010 (Figure 1). Our review here intends to highlight recent population-based research on host and viral correlates of HIV-1 VL set-point or its equivalent. For clarity and fair comparisons, studies assessing the relationship between host and/or viral factors on early set-point VL were selected according to two phenotypes, *i.e.;* set-point and chronic VLs as continuous or categorical endpoints. In addition, it was necessary to exclude studies dealing with children or youth (rare) or with small sample sizes (<100 HAART-naïve subjects). In the end, a total of 22 original research articles remained after four rounds of selection (Figure 1). Interpretation of these recent studies is relatively straightforward when supporting evidence from earlier reports is available. 

## 2. Host Genetics and Set-Point VL

### 2.1 Human Leukocyte Antigen (HLA) Class I and Class II Genes as Prominent Factors

HLA molecules mediate immune responses through multiple mechanisms, and their importance to effective immune control of HIV-1 infection has been well publicized in the past two decades [9,25,26,27]. Polymorphisms around the peptide-binding groove of HLA class I (HLA-I) and HLA-II molecules determine the specificity of cytotoxic T-lymphocyte responses (CTLs) and T-helper cell epitopes, respectively [28]. Direct interactions between HLA-I and killer cell immunoglobulin-like receptors (KIRs) can dictate natural killer (NK) cell function [29], which is further regulated by HLA leader peptides loaded to HLA-E [30,31]. These intertwined properties essential to both innate and adaptive immunity inevitably complicate the analysis of individual HLA alleles and certain functionally relevant residues or motifs shared by different alleles.

When individual HLA-I alleles are compared, new findings (Table 1) continue to support the notion that *HLA-A* and *HLA-C* alleles are less prominent than *HLA-B* alleles [32,33,34,35,36]. Specifically, studies have readily recognized HLA-B*13, B*14, B*18, B*27, B*35, B*44, B*45, B*53, B*57, B*58:01, B*58:02 and B*81  as distinct correlates of HIV-1 VL in several cohorts from Africa and North America [32,33,34,35,36]. Evidence for three *HLA-A* alleles (A*32, A*36, and A*74), two *HLA-C* alleles(C*08 and C*18), and one combination (HLA-A*30+HLA-C*03) is rather consistent with earlier observations, with HLA-A*74 being favorable (low VL) in native Africans and African-Americans [34,35,37,38,39]. Linkage disequilibrium (LD) between HLA-A*74 and HLA-B*57 may obscure the analysis of the former, but an independent contribution by A*74 was evident in a large sample size [39]. HLA-C*18 as a favorable allele needs further assessment, as it apparently tags two favorable *HLA-B* alleles, B*57:03 and B*81:01 [34,40]. The HLA-C*12—HLA-B*39 haplotype is another example of neighboring alleles that are hard to separate [33,35].

For HLA-II (Table 1), only two alleles have shown appreciable impact on set-point VL: HLA-DRB1*01:02 and HLA-DRB1*13:03 are associated with relatively high and low VL, respectively [34,41]. Of note, HLA-DRB1*01:02 was associated with high VL in a combined cohort of seroconverting patients (SCs) and seroprevalent patients (SPs) or in SPs alone [34]. In theory, SCs are more suitable for association analyses as few viral mutations are seen in early infection when set-point VL is measured. The relatively late effect of HLA-DRB1*01:02 (if confirmed) may reflect the delayed onset of high-affinity antibody responses mediated by HLA-II products. On the other hand, HLA-DRB1*13:03 is in moderate LD with HLA-B*57, but its association with low VL remained clear even when patients with HLA-B*57 were excluded [41].

When the mature HLA-B protein forms are inferred from *HLA-B* genotyping results, three amino acid residues at positions 67, 70, and 97 (Met^67^, Ser^70^ and Val^97 ^around the C and F pockets) seem to explain alleles (e.g.; B*57) associated with favorable outcomes (HIV-1 control) [42]. 

**Table 1:** Host Genetic Factors That Are Positively or Negatively Associated with HIV-1 Viral Load (VL) Set-Point or Assumed Set-point, as Reported in Recent Studies.^a^

Gene or gene cluster^b^	Allele or haplotype^c^	Ethnicity^d^	Impact on VL	Refs
Classical HLA class I genes: *HLA-A, HLA-B*, and *HLA-C*	A*32	AA	Favorable	[35]
	A*36	African	Unfavorable	[34, 35]
	A*74	AA, African	Favorable	[33,34,35,39]
	B*13	African	Favorable	[34]
	B*14	AA	Favorable	[35]
	B*18	African	Unfavorable	[33]
	B*27	Caucasian	Favorable	[32]
	B*35	Caucasian	Unfavorable	[32,35]
	B*44	African	Favorable	[36]
	B*45	AA, African	Unfavorable	[34,35]
	B*53	AA	Unfavorable	[35]
	B*57	AA, African, Caucasian	Favorable	[32,33,34,35,36,39,43]
	B*58:01	African	Favorable	[33,43]
	B*58:02	African	Unfavorable	[33,34]
	B*81	African	Favorable	[34]
	C*08	African	Favorable	[35]
	C*18	African	Favorable	[34,35]
	A*30+C*03	African	Favorable	[34]
	C*04:01-B*81:01	African	Favorable	[33]
	C*12-B*39	African	Favorable	[33,35]
	Homozygosity	AA and African	Unfavorable	[33,35]
*HLA-DRB1*	DRB1*01:02	African	Unfavorable	[34]
	DRB1*13:03	African	Favorable	[41]
Killer cell immunoglobulin-like receptor (KIR) genes	*KIR3DS1* copy no.	Caucasian	Favorable if ≥1 copy	[44]
	*KIR3DL1* copy no.	Caucasian	Favorable if ≥1 copy	[44]
*CCR5*	**Δ32** heterozygosity	Caucasian	Favorable	[45]
*CCR2-CCR5*	HHD/HHE	African	Unfavorable	[46]
	HHF*2 homozygosity	African	Favorable	[46]
*CCL3*	rs5029410 allele C	African	Favorable	[47]
*DC-SIGNR (CD209L)*	7 or 9 repeats of a 69-bp coding sequence	Asian (Chinese)	Unfavorable	[48]
Miscellaneous loci (sporadic SNPs)	rs2395029, allele C	Caucasian	Favorable	[45,49]
	rs9264942, allele G	Caucasian	Favorable	[45,49]

^a^ Four studies [50,51,52,53] with mostly negative results (not reaching statistical significance) are cited briefly in the text. ^b^ Organized by group and sorted by degree of popularity, *i.e.*; the number of studies meeting criteria (see Figure 1). ^c^ **Variants in bold** have shown consistency between studies conducted by different investigators. Certain amino acid residues may account for *HLA-B* allelic effects (e.g.; B*57 and B*81) [42,54], as discussed in the text. ^d^ AA=African American.

In African Americans, nonsynonymous single nucleotide polymorphism (SNPs) corresponding to HLA-B amino acid positions 63, 97, and 116 account for much of the effects attributable to the *HLA-B* locus [54]. However, HLA-B*44 alleles (Ser^67^, Asn^70^ and Arg^97^) that are favorable in native Africans did not conform to this newly recognized rule [36]. Similarity or difference in peptide-binding preferences alone may not fully capture the spectrum of concerted and evolving immune function that is essential to durable containment of HIV-1 infection [55]. 

Specific alleles and codon positions aside, HLA-I homozygosity (lack of diversity) has what appears to be an additive effect on set-point VL [33,35] (Table 1), probably by allowing rapid viral immune escape. Homozygosity is mostly restricted to common HLA-I alleles found in a given population, so its disadvantage may alternatively imply the advantage of rare or infrequent alleles to which viral adaptation is less likely to occur [56]. This concept of allele frequency-dependent influences on HIV-1 pathogenesis deserves further evaluation [35,38].

### 2.2 Killer Cell Immunoglobulin-like Receptor (KIR) Genes

KIR gene products are primarily expressed on natural killer (NK) cells to inhibit or activate cytotoxic activities, depending on the combination of receptor-ligand (HLA-B or HLA-C) pairing [53,57,58]. Just like their HLA ligands, KIR molecules are highly polymorphic in terms of gene content and allelic diversity. In the presence of HLA-B ligand Bw4-80I, the activating KIR3DS1 and inhibitory KIR3DL1 may delay HIV-1 disease progression (time to AIDS or death) [57,59]. The specific role of KIR-HLA interaction in the early course of HIV-1 infection is not obvious [60]. 

New evidence now suggests that *KIR3DS1* copy number variation is worth noting (Table 1). When HLA-Bw4-80I is present, one or more copies of *KIR3DS1* was associated with relatively low set-point VL even after statistical adjustments for other known factors in the KIR-HLA interaction pathway, including HLA-B*57, B*27, and B*35Px [44]. Two other recent studies found no association between *KIR3DL1*, *KIR3DS1,* or *KIR2DS4* and viral load [52,53]. Differences in methodology and *KIR3DS1* population frequencies may account for the lack of immediate consensus.

### 2.3 Chemokine Receptors and Ligand Genes

Several chemokine receptors, especially CCR5 and CCR2, act as HIV-1 co-receptors that facilitate viral entry into target cells. Neighboring *CCR2* and *CCR5* gene variants (haplotypes and diplotypes) have well-known relationships to HIV-1 transmission (initiation of infection) [61], but their role in established infection is not persuasive [25,62]. Heterozygosity for the 32-base-pair deletion in the *CCR5* gene open reading frame is of epidemiological importance to various populations [62,63,64,65], so is the amino acid substitution of valine to isoleucine at CCR2 residue 64 (64V/I). The *CCR2-CCR5* haplotypes tagged by *CCR5-Δ32* (HHG*2) and *CCR2-64I* (HHF*2) may act in concert to influence set-point VL in populations of European ancestry [62], but that combination (HHF*2/HHG*2) is too rare in other racial groups to be a worthy topic. Further work on various genes encoding CCR5 ligands (MIP-1α, MIP-1β, and CCL5/RANTES) often leads to inconsistent or conflicting observations [66]. 

Investigation of chemokine receptor and ligand genes is still active (Table 1). Translation of *CCR5-Δ32* to low set-point VL has gained new supporting evidence [45]. Modest advantage was also seen with HHF*2 homozygosity [46]. The HHD/HHE diplotype commonly seen in cohorts of African ancestry appeared to be unfavorable [46]. More recently, the minor allele C for SNP rs5029410 (in the *CCL3* gene) has been associated with low set-point VL [47], with a low probability of false discovery from multiple testing.

### 2.4 Other Miscellaneous Observations Based on Candidate Gene Approach

One study has revealed that *DC-SIGNR* (*CD209L*) genotypes can be associated with HIV-1 VL: the alleles encoding 7-repeat and 9-repeat isoforms appear to be unfavorable [48] (Table 1). The number of 23-amino acid repeats in the DC-SIGNR protein ranges from three to nine [67], and the reported associations can be attributed to two isoform combinations, 5/7 and 7/9. Biologically, DC-SIGNR and DC-SIGN are transmembrane receptors on dendritic cells that help ferry HIV-1 virions to tissues enriched with CD4^+^ T-cells [68]. Earlier work has shown some evidence about a possible distinction between the seven- or nine-repeat isoforms and others [67].

### 2.5 Results From Genome-wide Association Studies (GWAS)

GWAS provide a hypothesis-free approach to identifying genes or SNPs of epidemiological importance. Multiple GWAS have consistently pointed to two SNPs as markers of effective immune control during HIV-1 infection. The first, rs2395029, is mapped to the *HCP5* pseudogene. The second, rs9264942, is located about 35-kb upstream of *HLA-C* [69,70,71,72,73,74,75,76]. In Caucasians, these SNPs effectively tag HLA-B*57:01 and a microRNA target site polymorphism in *HLA-C* 3’ untranslated region (UTR), respectively [69,77]. Other HLA-I alleles can be involved as well [71,72,78,79]. 

Variants defined by rs2395029 and rs9264942 are highlighted in two new studies [45,49] (Table 1). Separate analysis of SCs and SPs is considered useful as the effect sizes for many individual SNPs can vary greatly between SCs and SPs [49]. Two other GWAS based on African-Americans and native Africans failed to identify any SNPs with genome-wide statistical significance [50,51]. In the African-American cohort, the top 10 SNPs of interest are all within the human major histocompatibility complex (MHC) [50]. The SNP (rs2523608) with the best association signal (Table 1) is actually in LD with HLA-B*57:03 (a favorable allele). In analysis of native Africans [51], the number one SNP of interest (rs13111989) is beyond the MHC region (Table 1). 

## 3. Viral Genetics and HIV-1 Set-point VL

### 3.1 HIV-1 Genotype

Epidemiologists and virologists are acutely aware of the evidence that defective viruses might partially explain spontaneous HIV-1 control, as seen in the strings of patients infected by a single blood donor in Sydney, Australia [80,81]. The ability of such viruses to cause sexual transmission (an inefficient process) is unclear, but recent analyses of 134 native Africans with sexually transmitted primary HIV-1 infection [36] did reveal that acute-phase VL can be low (<2,000 copies/mL) in a small proportion (~6.7%) of SCs. Direct experimental evidence is still elusive as infectious viruses are hard to recover from these subjects. Conversely, however, SCs with set-point VL below 50 copies/mL can have measurable acute-phase VL (>10,000 copies/mL) [36]. Other investigators have also come across rare cases where elite control was possible even when highly pathogenic viruses from clinical AIDS patients were transmitted [82]. 

**Table 2:** Viral Markers That Are Associated with HIV-1 Set-Point Viral Load (VL), as Reported in Recent Studies.

Viral factor	Measurement	Impact on set-point VL	Refs
Heritability	Transmission source partner (TSP) VL	TSP VL correlates with set-point VL in linked recipients	[83]
	Genetic distance on phylogenetic tree	High heritability in set-point VL, from one infection to the next	[84]

Following and verifying HIV-1 transmission chains are not easily done, but the assessment of donor and recipient VL can be useful [85]. New results from analyses of linked transmission pairs (Table 2) support a modest linear relationship between donor VL (chronic) and recipient set-point VL [83]. In a second study, genetic distances between viral sequences correlate with differences in VL [84], suggesting that viral genotypes should be considered during the search for quantitative trait loci.

### 3.2 Interaction of Host and Viral Genetic Factors

To properly dissect out factors (host or viral) with the greatest influence on HIV-1 evolution and viral load, models will need to simultaneously consider host and viral dynamics [83,85,86]. Among three closely related *HLA-B* allelic products examined in this context [43], HLA-B*57:03 appears to target four p24 Gag epitopes (ISW9, KF11, TW10, and QW9), but HLA-B*57:02 and HLA-B*58:01 only target three and two of them, respectively [43]. Conceivably, these allelic forms can impose differential selection pressure on the viral genome. In the end, the causal factors of viral fitness can lie in the host and in the transmitted virus. 

## 4. Methodological Challenges

### 4.1 Variations in Calculation of Set-point VL

Despite its wide use, there is still no standard method for determining HIV-1 set-point VL, with multiple methods having been used rather randomly [87]. When a single RNA measurement is treated as the set-point [16,71,88], the timing of such measurement can vary greatly: (i) the visit after the first seropositive visit, (ii) visit at least three months after the estimated date of infection (EDI), (iii) visit at least six months after the EDI. Others prefer to use data from several visits [12,89], in favor of methods that calculate the VL phenotype as the average or as the median of multiple VL points within specific intervals of infection [87]. Those with more advanced statistical skills simply test repeated VL measurements in mixed models [36,78], but asymmetry in data structure (total visits and visit intervals) can be an issue. Decision to exclude patients with insufficient data can be a sticky business.

### 4.2 Early Chronic Phase *Versus* Chronic Phase

Viral adaptation to the host microenvironment, including protective immune responses, is a gradual process. VLs taken during the early and later course of infection can possess similar traits for very different reasons [24,34,49], so findings are not directly comparable when the duration of infection is unknown. As most studies have already missed the early course of infection [65], the literature is likely most relevant to chronic infection when opportunistic infections (OIs) may complicate the analysis [33,34,35]. The OIs can be disparate in exposure, tissue compartments, and geography, but they are rarely captured in analysis of HIV-1 VL readouts.

### 4.3 Changes in Set-point VL over Calendar Time

Several studies have noted an increase in set-point VL over time [45,90,91,92], while others disagree [93,94,95]. A large meta-analysis pooling results from prior studies of seroincident patients found a trend for a rising VL set-point over time [96]. Assuming that widespread viral adaptation does occur [45,96,97], one can envision that the timing of the AIDS epidemic in different populations can be critical. In an European population, pre-2003 set-point VL appeared to differ from post-2003 VL in SCs [91], accompanied by a loss of host genetic advantage conferred by *CCR5-Δ32* and other prominent factors (e.g.; rs2395029/B*57:01) [45]. Likewise, patients with HLA-B*51 before and after 2001 differed in their VLs [98], which is consistent with the hypothesis that specific CTL escape mutations induced by HLA-mediated immunity can reach fixation when these mutations have no apparent fitness costs [99]. Finding the tipping point for adapted *versus* unadapted viruses in each population is obviously another sticky business. 

### 4.4 Other Potential Confounders

Cofactors not routinely considered in analysis of HIV-1 VL can be quite obvious. For example, age and gender are known to influence VL [37], but they are infrequently seen in reported statistical models. Other less obvious factors can range from viral subtypes and its segregation with certain racial backgrounds [100,101] to differential distribution of important genetic variations (e.g.; *CCR5-Δ32* and HLA-B*27) or the techniques used for defining them. Future studies will clearly need to apply multivariable models to carefully consider covariates and potential confounders. Composite scores based on all known factors may offer a temporary solution to simplifying the data analysis process [13,102], although individual factors may not have equally additive effects on HIV-1 VL.

## 5. Conclusions

HIV-1 viremia is an informative quantitative trait that varies at the individual and population levels. While many studies have attempted to sort out the quantitative trait loci, lack of clear consensus hints at various problems with study design and data analysis.

Factors important to VL can lie in the host and viral genomes. As viral evolution shaped by host immune responses become more and more predictable, fine-mapping of viral and host genetics can begin to allow a fair assessment of primary and secondary factors for transformative research. In other words, an open-minded research question is not whether host factors predominate over viral factors or vice versa, the two are so intertwined that their constant interactions in distinct individuals and populations collectively dictate the landscape of viral dynamics. The ultimate challenge (and goal) is to properly integrate comprehensive data on host and viral characteristics. The need for such approach is urgent, as datasets generated by high-throughput techniques will become overwhelmingly complex.

## References

[1] Gottlieb G.S., Heath L., Nickle D.C., Wong K.G., Leach S.E., Jacobs B., Gezahegne S., van 't Wout A.B., Jacobson L.P., Margolick J.B. (2008). HIV-1 variation before seroconversion in men who have sex with men: analysis of acute/early HIV infection in the multicenter AIDS cohort study. J Infect Dis.

[2] Keele B.F., Giorgi E.E., Salazar-Gonzalez J.F., Decker J.M., Pham K.T., Salazar M.G., Sun C., Grayson T., Wang S., Li H. (2008). Identification and characterization of transmitted and early founder virus envelopes in primary HIV-1 infection. Proc Natl Acad Sci U S A.

[3] Abrahams M.R., Anderson J.A., Giorgi E.E., Seoighe C., Mlisana K., Ping L.H., Athreya G.S., Treurnicht F.K., Keele B.F., Wood N. (2009). Quantitating the multiplicity of infection with human immunodeficiency virus type 1 subtype C reveals a non-poisson distribution of transmitted variants. J Virol.

[4] Haaland R.E., Hawkins P.A., Salazar-Gonzalez J., Johnson A., Tichacek A., Karita E., Manigart O., Mulenga J., Keele B.F., Shaw G.M. (2009). Inflammatory genital infections mitigate a severe genetic bottleneck in heterosexual transmission of subtype A and C HIV-1. PLoS Pathog.

[5] Kearney M., Maldarelli F., Shao W., Margolick J.B., Daar E.S., Mellors J.W., Rao V., Coffin J.M., Palmer S. (2009). Human immunodeficiency virus type 1 population genetics and adaptation in newly infected individuals. J Virol.

[6] Walker B.D., Korber B.T. (2001). Immune control of HIV: the obstacles of HLA and viral diversity. Nat Immunol.

[7] Korber B., Muldoon M., Theiler J., Gao F., Gupta R., Lapedes A., Hahn B.H., Wolinsky S., Bhattacharya T. (2000). Timing the ancestor of the HIV-1 pandemic strains. Science.

[8] Tebit D.M., Arts E.J. (2011). Tracking a century of global expansion and evolution of HIV to drive understanding and to combat disease. Lancet Infect Dis.

[9] Boutwell C.L., Rolland M.M., Herbeck J.T., Mullins J.I., Allen T.M. (2010). Viral evolution and escape during acute HIV-1 infection. J Infect Dis.

[10] Mellors J.W., Rinaldo C.R.J., Gupta P., White R.M., Todd J.A., Kingsley L.A. (1996). Prognosis in HIV-1 infection predicted by the quantity of virus in plasma. Science.

[11] Mellors J.W., Muñoz A., Giorgi J.V., Margolick J.B., Tassoni C.J., Gupta P., Kingsley L.A., Todd J.A., Saah A.J., Detels R. (1997). Plasma viral load and CD4+ lymphocytes as prognostic markers of HIV-1 infection. Ann Intern Med.

[12] de Wolf F., Spijkerman I., Schellekens P.T., Langendam M., Kuiken C., Bakker M., Roos M., Coutinho R., Miedema F., Goudsmit J. (1997). AIDS prognosis based on HIV-1 RNA, CD4+ T-cell count and function: markers with reciprocal predictive value over time after seroconversion. AIDS.

[13] Mann D.L., Garner R.P., Dayoff D.E., Cao K., Fernandez-Vina M.A., Davis C., Aronson N., Ruiz N., Birx D.L., Michael N.L. (1998). Major histocompatibility complex genotype is associated with disease progression and virus load levels in a cohort of human immunodeficiency virus type 1-infected Caucasians and African Americans. J Infect Dis.

[14] Lyles R.H., Muñoz A., Yamashita T.E., Bazmi H., Detels R., Rinaldo C.R., Margolick J.B., Phair J.P., Mellors J.W. (2000). Natural history of human immunodeficiency virus type 1 viremia after seroconversion and proximal to AIDS in a large cohort of homosexual men. Multicenter AIDS Cohort Study. J Infect Dis.

[15] Gottlieb G.S., Sow P.S., Hawes S.E., Ndoye I., Redman M., Coll-Seck A.M., Faye-Niang M.A., Diop A., Kuypers J.M., Critchlow C.W. (2002). Equal plasma viral loads predict a similar rate of CD4+ T cell decline in human immunodeficiency virus (HIV) type 1- and HIV-2-infected individuals from Senegal, West Africa. J Infect Dis.

[16] Mellors J.W., Margolick J.B., Phair J.P., Rinaldo C.R., Detels R., Jacobson L.P., Muñoz A. (2007). Prognostic value of HIV-1 RNA, CD4 cell count, and CD4 Cell count slope for progression to AIDS and death in untreated HIV-1 infection. JAMA.

[17] Lavreys L., Baeten J.M., Chohan V., McClelland R.S., Hassan W.M., Richardson B.A., Mandaliya K., Ndinya-Achola J.O., Overbaugh J. (2006). Higher set point plasma viral load and more-severe acute HIV type 1 (HIV-1) illness predict mortality among high-risk HIV-1-infected African women. Clin Infect Dis.

[18] Quinn T.C., Wawer M.J., Sewankambo N., Serwadda D., Li C., Wabwire-Mangen F., Meehan M.O., Lutalo T., Gray R.H. (2000). Viral load and heterosexual transmission of human immunodeficiency virus type 1. Rakai Project Study Group. N Eng J Med.

[19] Fideli U.S., Allen S.A., Musonda R., Trask S., Hahn B.H., Weiss H., Mulenga J., Kasolo F., Vermund S.H., Aldrovandi G.M. (2001). Virologic and immunologic determinants of heterosexual transmission of human immunodeficiency virus type 1 in Africa. AIDS Res Hum Retroviruses.

[20] Daar E.S., Moudgil T., Meyer R.D., Ho D.D. (1991). Transient high levels of viremia in patients with primary HIV-1 infection. N Engl J Med.

[21] Geskus R.B., Prins M., Hubert J.B., Miedema F., Berkhout B., Rouzioux C., Delfraissy J.F., Meyer L. (2007). The HIV RNA setpoint theory revisited. Retrovirology.

[22] National Institute of Allergy and Infectious Diseases (2008). What are HIV and AIDS?. HIV/AIDS.

[23] Egger M., May M., Chêne G., Phillips A.N., Ledergerber B., Dabis F., Costagliola D., D'Arminio Monforte A., de Wolf F., Reiss P. (2002). Prognosis of HIV-1-infected patients starting highly active antiretroviral therapy: a collaborative analysis of prospective studies. Lancet.

[24] Langford S.E., Ananworanich J., Cooper D.A. (2007). Predictors of disease progression in HIV infection: a review. AIDS Res Ther.

[25] Tang J., Kaslow R.A. (2003). The impact of host genetics on HIV infection and disease progression in the era of highly active antiretroviral therapy. AIDS.

[26] Carrington M., O'Brien S.J. (2003). The influence of HLA genotype on AIDS. Annu Rev Med.

[27] Streeck H., Jolin J.S., Qi Y., Yassine-Diab B., Johnson R.C., Kwon D.S., Addo M.M., Brumme C., Routy J.P., Little S. (2009). Human immunodeficiency virus type 1-specific CD8+ T-cell responses during primary infection are major determinants of the viral set point and loss of CD4+ T cells. J Virol.

[28] Horton R., Wilming L., Rand V., Lovering R.C., Bruford E.A., Khodiyar V.K., Lush M.J., Povey S., Talbot C.C.J., Wright M.W. (2004). Gene map of the extended human MHC. Nat Rev Genet.

[29] Carrington M., Martin M.P., van Bergen J. (2008). KIR-HLA intercourse in HIV disease. Trends Microbiol.

[30] O'Callaghan C.A., Bell J.I. (1998). Structure and function of the human MHC class Ib molecules HLA-E, HLA-F and HLA-G. Immunol Rev.

[31] Yunis E.J., Romero V., Diaz-Giffero F., Zuniga J., Koka P. (2007). Natural killer cell receptor NKG2A/HLA-E interaction dependent differential thymopoiesis of hematopoietic progenitor cells influences the outcome of HIV infection. J Stem Cells.

[32] Gao X., O'Brien T.R., Welzel T.M., Marti D., Qi Y., Goedert J.J., Phair J., Pfeiffer R., Carrington M. (2010). HLA-B alleles associate consistently with HIV heterosexual transmission, viral load, and progression to AIDS, but not to susceptability to infectin. AIDS.

[33] Leslie A., Matthews P.C., Listgarten J., Carlson J.M., Kadie C., Ndung'u T., Brander C., Coovadia H., Walker B.D., Heckerman D. (2010). Additive contribution of HLA class I alleles in the immune control of HIV-1 infection. J Virol.

[34] Tang J., Malhotra R., Song W., Brill I., Hu L., Farmer P.K., Mulenga J., Allen S., Hunter E., Kaslow R.A. (2010). Human leukocyte antigens and HIV type 1 viral load in early and chronic infections: predominance of evolving relationships. PLoS One.

[35] Lazaryan A., Song W., Lobashevsky E., Tang J., Shrestha S., Zhang K., McNicholl J.M., Gardner L.I., Wilson C.M., Klein R.S. (2011). The influence of human leukocyte antigen class I alleles and their population frequencies on human immunodeficiency virus type 1 control among African Americans. Hum Immunol.

[36] Tang J., Cormier E., Gilmour J., Price M.A., Prentice H.A., Song W., Kamali A., Karita E., Lakhi S., Sanders E.J. (2011). Human leukocyte antigen variants B*44 and B*57 are consistently favorable during two distinct phases of primary HIV-1 infection in sub-Saharan Africans with several viral subtypes. J Virol.

[37] Tang J., Tang S., Lobashevsky E., Myracle A.D., Fideli U., Aldrovandi G., Allen S., Musonda R., Kaslow R.A. (2002). Zambia-UAB HIV Research Project. Favorable and unfavorable HLA class I alleles and haplotypes in Zambians predominantly infected with clade C human immunodeficiency virus type 1. J Virol.

[38] Trachtenberg E., Korber B., Sollars C., Kepler T.B., Hraber P.T., Hayes E., Funkhouser R., Fugate M., Theiler J., Hsu Y.S. (2003). Advantage of rare HLA supertype in HIV disease progression. Nat Med.

[39] Matthews P.C., Adland E., Listgarten J., Leslie A., Mkhwanazi N., Carlson J.M., Harndahl M., Stryhn A., Payne R.P., Ogwu A. (2011). HLA-A*7401-mediated control of HIV viremia is independent of its linkage disequilibrium with HLA-B*5703. J Immunol.

[40] Tang J., Shao W., Yoo Y.J., Brill I., Mulenga J., Allen S., Hunter E., Kaslow R.A. (2008). Human leukocyte antigen class I genotypes in relation to heterosexual HIV type 1 transmission within discordant couples. J Immunol.

[41] Julg B., Moodley E.S., Qi Y., Ramduth D., Reddy S., Mncube Z., Gao X., Goulder P.J., Detels R., Ndung'u T. (2011). Possession of HLA class II DRB1*1303 associates with reduced viral loads in chronic HIV-1 clade C and B infection. J Infect Dis.

[42] Pereyra F., International HIV Controllers Study (2010). The major genetic determinants of HIV-1 control affect HLA class I peptide presentation. Science.

[43] Kloverpris H.N., Stryhn A., Harndahl M., van der Stok M., Payne R.P., Matthews P.C., Chen F., Riddell L., Walker B.D., Ndung'u T. (2012). HLA-B*57 micropolymorphism shapes HLA allele-specific epitope immunogenicity, selection pressure, and HIV immune control. J Virol.

[44] Pelak K., Need A.C., Fellay J., Shianna K.V., Feng S., Urban T.J., Ge D., De Luca A., Martinez-Picado J., Wolinsky S.M. (2011). Copy number variation of KIR genes influences HIV-1 control. PLoS Biol.

[45] van Manen D., Gras L., Boeser-Nunnink B.D., van Sighem A.I., Mangas Ruiz M.M., Harskamp A.M., Steingrover R., Prins J.M., de Wolf F., van't Wout A.B. (2011). Rising HIV-1 viral load set point at a population level coincides with a fading impact of host genetic factors on HIV-1 control. AIDS.

[46] Malhotra R., Hu L., Song W., Brill I., Mulenga J., Allen S., Hunter E., Shrestha S., Tang J., Kaslow R.A. (2011). Association of chemokine receptor gene (CCR2-CCR5) haplotypes with acquisition and control of HIV-1 infection in Zambians. Retrovirology.

[47] Hu L., Song W., Brill I., Mulenga J., Allen S., Hunter E., Shrestha S., Tang J., Kaslow R.A. (2012). Genetic variations and heterosexual HIV-1 infection: analysis of clustered genes encoding CC-motif chemokine ligands. Genes Immun.

[48] Xu L., Li Q., Ye H., Zhang Q., Chen H., Huang F., Chen R., Zhou R., Zhou W., Xia P. (2010). The nine-repeat DC-SIGNR isoform is associated with increased HIV-RNA loads and HIV sexual transmission. J Clin Immunol.

[49] Evangelou E., Fellay J., Colombo S., Martinez-Picado J., Obel N., Goldstein D.B., Telenti A., Ioannidis J.P. (2011). Impact of phenotype definition on genome-wide association signals: empirical evaluation in human immunodeficiency virus type 1 infection. Am J Epidemiol.

[50] Pelak K., Goldstein D.B., Walley N.M., Fellay J., Ge D., Shianna K.V., Gumbs C., Gao X., Maia J.M., Cronin K.D. (2010). Host determinants of HIV-1 control in African Americans. J Infect Dis.

[51] Lingappa J.R., Petrovski S., Kahle E., Fellay J., Shianna K., McElrath M.J., Thomas K.K., Baeten J.M., Celum C., Wald A. (2011). Genomewide association study for determinants of HIV-1 acquisition and viral set point in HIV-1 serodiscordant couples with quantified virus exposure. PLoS One.

[52] Silva E.M., Acosta A.X., Santos E.J., Netto E.M., Lemaire D.C., Oliveira A.S., Barbosa C.M., Bendicho M.T., Galvão-Castro B., Brites C. (2010). HLA-Bw4-B*57 and Cw*18 alleles are associated with plasma viral load modulation in HIV-1 infected individuals in Salvador, Brazil. Braz J Infect Dis.

[53] Merino A., Malhotra R., Morton M., Mulenga J., Allen S., Hunter E., Tang J., Kaslow R.A. (2011). Impact of a functional KIR2DS4 allele on heterosexual HIV-1 transmission among discordant Zambian couples. J Infect Dis.

[54] McLaren P.J., Ripke S., Pelak K., Weintrob A.C., Patsopoulos N.A., Jia X., Erlich R.L., Lennon N.J., Kadie C.M., Heckerman D. (2012). Fine-mapping classical HLA variation associated with durable host control of HIV-1 infection in African Americans. Hum Mol Genet.

[55] Koup R.A., Graham B.S., Douek D.C. (2011). The quest for a T cell-based immune correlate of protection against HIV: a story of trials and errors. Nat Rev Immunol.

[56] Rousseau C.M., Daniels M.G., Carlson J.M., Kadie C., Crawford H., Prendergast A., Matthews P., Payne R., Rolland M., Raugi D.N. (2008). HLA class I-driven evolution of human immunodeficiency virus type 1 subtype c proteome: immune escape and viral load. J Virol.

[57] Qi Y., Martin M.P., Gao X., Jacobson L., Goedert J.J., Buchbinder S., Kirk G.D., O'Brien S.J., Trowsdale J., Carrington M. (2006). KIR/HLA pleiotropism: protection against both HIV and opportunistic infections. PLoS Pathog.

[58] Vilches C., Parham P. (2002). KIR: diverse, rapidly evolving receptors of innate and adaptive immunity. Annu Rev Immunol.

[59] Martin M.P., Qi Y., Gao X., Yamada E., Martin J.N., Pereyra F., Colombo S., Brown E.E., Shupert W.L., Phair J. (2007). Innate partnership of HLA-B and KIR3DL1 subtypes against HIV-1. Nat Genet.

[60] Gaudieri S., DeSantis D., McKinnon E., Moore C., Nolan D., Witt C.S., Mallal S.A., Christiansen F.T. (2005). Killer immunoglobulin-like receptors and HLA act both independently and synergistically to modify HIV disease progression. Genes Immun.

[61] Kaslow R.A., Dorak T., Tang J.J. (2005). Influence of Host Genetic Variation on Susceptibility to HIV Type 1 Infection. J Infect Dis.

[62] Tang J., Shelton B., Makhatadze N.J., Zhang Y., Schaen M., Louie L.G., Goedert J.J., Seaberg E.C., Margolick J.B., Mellors J. (2002). Distribution of chemokine receptor CCR2 and CCR5 genotypes and their relative contribution to human immunodeficiency virus type 1 (HIV-1) seroconversion, early HIV-1 RNA concentration in plasma, and later disease progression. J Virol.

[63] Katzenstein T.L., Eugen-Olsen J., Hofmann B., Benfield T., Pedersen C., Iversen A.K., Sørensen A.M., Garred P., Koppelhus U., Svejgaard A. (1997). HIV-infected individuals with the CCR delta32/CCR5 genotype have lower HIV RNA levels and higher CD4 cell counts in the early years of the infection than do patients with the wild type. Copenhagen AIDS Cohort Study Group. J Acquir Immune Defic Syndr Hum Retrovirol.

[64] Meyer L., Magierowska M., Hubert J.B., Rouzioux C., Deveau C., Sanson F., Debre P., Delfraissy J.F., Theodorou I. (1997). Early protective effect of CCR-5 delta 32 heterozygosity on HIV-1 disease progression: relationship with viral load. The SEROCO Study Group. AIDS.

[65] Ioannidis J.P., Rosenberg P.S., Goedert J.J., Ashton L.J., Benfield T.L., Buchbinder S.P., Coutinho R.A., Eugen-Olsen J., Gallart T., Katzenstein T.L. (2001). Effects of CCR5-Delta32, CCR2-64I, and SDF-1 3'A alleles on HIV-1 disease progression: An international meta-analysis of individual-patient data. Ann Intern Med.

[66] Shrestha S., Tang J., Kaslow R.A. (2009). Gene copy number: learning to count past two. Nat Med.

[67] Liu H., Carrington M., Wang C., Holte S., Lee J., Greene B., Hladik F., Koelle D.M., Wald A., Kurosawa K. (2006). Repeat-region polymorphisms in the gene for the dendritic cell-specific intercellular adhesion molecule-3-grabbing nonintegrin-related molecule: effects on HIV-1 susceptibility. J Infect Dis.

[68] Geijtenbeek T.B., van Duijnhoven G.C., van Vliet S.J., Krieger E., Vriend G., Figdor C.G., van Kooyk Y. (2002). Identification of different binding sites in the dendritic cell-specific receptor DC-SIGN for intercellular adhesion molecule 3 and HIV-1. J Biol Chem.

[69] Fellay J., Shianna K.V., Ge D., Colombo S., Ledergerber B., Weale M., Zhang K., Gumbs C., Castagna A., Cossarizza A. (2007). A whole-genome association study of major determinants for host control of HIV-1. Science.

[70] Dalmasso C., Carpentier W., Meyer L., Rouzioux C., Goujard C., Chaix M.L., Lambotte O., Avettand-Fenoel V., Le Clerc S., de Senneville L.D. (2008). Distinct genetic loci control plasma HIV-RNA and cellular HIV-DNA levels in HIV-1 infection: the ANRS genome wide association 01 study. PLoS One.

[71] Catano G., Kulkarni H., He W., Marconi V.C., Agan B.K., Landrum M., Anderson S., Delmar J., Telles V., Song L. (2008). HIV-1 disease-influencing effects associated with *ZNRD1*, *HCP5* and *HLA-C* alleles are attributable mainly to either *HLA-A10* or *HLA-B*57* alleles. PLoS One.

[72] Fellay J., Ge D., Shianna K.V., Colombo S., Ledergerber B., Cirulli E.T., Urban T.J., Zhang K., Gumbs C., Smith J.P. (2009). Common genetic variation and the control of HIV-1 in humans. PLoS Genet.

[73] Thomas R., Apps R., Qi Y., Gao X., Male V., O'hUiqin C., O'Conner G., Ge D., Fellay J., Martin J.N. (2009). HLA-C cell surface expression and control of HIV/AIDS correlate with a variant upstream of HLA-C. Nat Genet.

[74] van Manen D., Kootstra N.A., Boeser-Nunnink B., Handulle M.A.M., van't Wout A.B., Schuitemaker H. (2009). Association of HLA-C and HCP5 gene regions with the clinical course of HIV-1 infection. AIDS.

[75] Guergnon J., Theodorou I. (2011). What did we learn on host's genetics by studying large cohorts of HIV-1-infected patients in the genome-wide association era?. Current Opinion in HIV and AIDS.

[76] Aouizerat B.E., Pearce C.L., Miaskowski C. (2011). The search for host genetic factors of HIV/AIDS pathogenesis in the post-genome era: progress to date and new avenues for discovery. Curr HIV/AIDS Rep.

[77] Kulkarni S., Savan R., Qi Y., Gao X., Yuki Y., Bass S.E., Martin M.P., Hunt P., Deeks S.G., Telenti A. (2011). Differential microRNA regulation of HLA-C expression and its association with HIV control. Nature.

[78] Shrestha S., Aissani B., Song W., Wilson C.M., Kaslow R.A., Tang J. (2009). Host genetics and HIV-1 viral load set-point in African Americans. AIDS.

[79] Trachtenberg E., Bhattacharya T., Ladner M., Phair J., Erlich H., Wolinsky S. (2009). The HLA-B/C haplotype block contains major determinants for host control of HIV. Genes Immun.

[80] Learmont J.C., Geczy A.F., Mills J., Ashton L.J., Raynes-Greenow C.H., Garsia R.J., Dyer W.B., McIntyre L., Oelrichs R.B., Rhodes D.I. (1999). Immunologic and virologic status after 14 to 18 years of infection with an attenuated strain of HIV-1. A report from the Sydney Blood Bank Cohort. N Engl J Med.

[81] Zaunders J., Dyer W.B., Churchill M. (2011). The Sydney Blood Bank Cohort: implications for viral fitness as a cause of elite control. Curr Opin HIV AIDS.

[82] Bailey J.R., O'Connell K., Yang H.C., Han Y., Xu J., Jilek B., Williams T.M., Ray S.C., Siliciano R.F., Blankson J.N. (2008). Transmission of human immunodeficiency virus type 1 from a patient who developed AIDS to an elite suppressor. J Virol.

[83] Hollingsworth T.D., Laeyendecker O., Shirreff G., Donnelly C.A., Serwadda D., Wawer M.J., Kiwanuka N., Nalugoda F., Collinson-Streng A., Ssempijja V. (2010). HIV-1 transmitting couples have similar viral load set-points in Rakai, Uganda. PLoS Pathog.

[84] Alizon S., von Wyl V., Stadler T., Kouyos R.D., Yerly S., Hirschel B., Boni J., Shah C., Klimkait T., Furrer H. (2010). Phylogenetic approach reveals that virus genotype largely determines HIV set-point viral load. PLoS Pathog.

[85] Tang J., Tang S., Lobashevsky E., Zulu I., Aldrovandi G., Allen S., Kaslow R.A. (2004). Zambia-UAB HIV Research Project. HLA allele sharing and HIV type 1 viremia in seroconverting Zambians with known transmitting partners. AIDS Res Hum Retroviruses.

[86] Novitsky V., Gilbert P., Peter T., McLane M.F., Gaolekwe S., Rybak N., Thior I., Ndung'u T., Marlink R., Lee T.H. (2003). Association between virus-specific T-cell responses and plasma viral load in human immunodeficiency virus type 1 subtype C infection. J Virol.

[87] Mei Y., Wang L., Holte S.E. (2008). A comparison of methods for determining HIV viral set point. Stat Med.

[88] Mellors J.W., Kingsley L.A., Rinaldo C.R.J., Todd J.A., Hoo B.S., Kokka R.P., Gupta P. (1995). Quantitation of HIV-1 RNA in plasma predicts outcome after seroconversion. Ann Intern Med.

[89] Hubert J.B., Burgard M., Dussaix E., Tamalet C., Deveau C., Le Chenadec J., Chaix M.L., Marchadier E., Vildé J.L., Delfraissy J.F. (2000). Natural history of serum HIV-1 RNA levels in 330 patients with a known date of infection. The SEROCO Study Group. AIDS.

[90] Dorrucci M., Rezza G., Porter K., Phillips A., Concerted Action on Seroconversion to AIDS and Death in Europe Collaboration (2007). Temporal trends in postseroconversion CD4 cell count and HIV load: the Concerted Action on Seroconversion to AIDS and Death in Europe Collaboration, 1985-2002. J Infect Dis.

[91] Gras L., Jurriaans S., Bakker M., van Sighem A., Bezemer D., Fraser C., Lange J., Prins J.M., Berkhout B., de Wolf F. (2009). Viral load levels measured at set-point have risen over the last decade of the HIV epidemic in the Netherlands. PLoS One.

[92] Müller V., Maggiolo F., Suter F., Ladisa N., De Luca A., Antinori A., Sighinolfi L., Quiros-Roldan E., Carosi G., Torti C. (2009). Increasing clinical virulence in two decades of the Italian HIV epidemic. PLoS One.

[93] Müller V., Ledergerber B., Perrin L., Klimkait T., Furrer H., Telenti A., Bernasconi E., Vernazza P., Günthard H.F., Bonhoeffer S. (2006). Stable virulence levels in the HIV epidemic of Switzerland over two decades. AIDS.

[94] Potard V., Weiss L., Lamontagne F., Rouveix E., Beck-Wirth G., Drogoul-Vey M.P., Souala M.F., Costagliola D., French Hospital Database on HIV ANRS CO4 (2009). Trends in post-infection CD4 cell counts and plasma HIV-1 RNA levels in HIV-1-infected patients in France between 1997 and 2005. J Acquir Immune Defic Syndr.

[95] Troude P., Chaix M.L., Tran L., Deveau C., Seng R., Delfraissy J.F., Rouzioux C., Goujard C., Meyer L., ANRS Primo Cohort (2009). No evidence of a change in HIV-1 virulence since 1996 in France. AIDS.

[96] Herbeck J.T., Muller V., Maust B.S., Ledergerber B., Torti C., Di Giambenedetto S., Gras L., Gunthard H.F., Jacobson L.P., Mullins J.I. (2012). Is the virulence of HIV changing? A meta-analysis of trends in prognostic markers of HIV disease progression and transmission. AIDS.

[97] Moore C.B., John M., James I.R., Christiansen F.T., Witt C.S., Mallal S. (2002). Evidence of HIV-1 adaptation to HLA-restricted immune responses at a population level. Science.

[98] Koga M., Kawana-Tachikawa A., Heckerman D., Odawara T., Nakamura H., Koibuchi T., Fujii T., Miura T., Iwamoto A. (2010). Changes in impact of HLA class I allele expression on HIV-1 plasma virus loads at a population level over time. Microbiol Immunol.

[99] Kawashima Y., Pfafferott K., Frater J., Matthews P., Payne R., Addo M., Gatanaga H., Fujiwara M., Hachiya A., Koizumi H. (2009). Adaptation of HIV-1 to human leukocyte antigen class I. Nature.

[100] Peeters M. (2001). The genetic variability of HIV-1 and its implications. Transfus Clin Biol.

[101] Eberle J., Gurtler L. (2012). HIV types, groups, subtypes and recombinant forms: errors in replication, selection pressure and quasispecies. Intervirology.

[102] Saah A.J., Hoover D.R., Weng S., Carrington M., Mellors J., Rinaldo C.R.J., Mann D., Apple R., Phair J.P., Detels R. (1998). Association of HLA profiles with early plasma viral load, CD4+ cell count and rate of progression to AIDS following acute HIV-1 infection. Multicenter AIDS Cohort Study. AIDS.

